# Disruption of the *pdhB* Pyruvate Dehydrogenase Gene Affects Colony Morphology, *In Vitro* Growth and Cell Invasiveness of *Mycoplasma agalactiae*

**DOI:** 10.1371/journal.pone.0119706

**Published:** 2015-03-23

**Authors:** Shivanand Hegde, Renate Rosengarten, Rohini Chopra-Dewasthaly

**Affiliations:** Division of Clinical Microbiology and Infection Biology, Institute of Bacteriology, Mycology and Hygiene, Department of Pathobiology, University of Veterinary Medicine Vienna, Vienna, Austria; Université Paris Descartes, FRANCE

## Abstract

The utilization of available substrates, the metabolic potential and the growth rates of bacteria can play significant roles in their pathogenicity. This study concentrates on *Mycoplasma agalactiae*, which causes significant economic losses through its contribution to contagious agalactia in small ruminants by as yet unknown mechanisms. This lack of knowledge is primarily due to its fastidious growth requirements and the scarcity of genetic tools available for its manipulation and analysis. Transposon mutagenesis of *M*. *agalactiae* type strain PG2 resulted in several disruptions throughout the genome. A mutant defective in growth in vitro was found to have a transposon insertion in the *pdhB* gene, which encodes a component of the pyruvate dehydrogenase complex. This growth difference was quite significant during the actively dividing logarithmic phase but a gradual recovery was observed as the cells approached stationary phase. The mutant also exhibited a different and smaller colony morphology compared to the wild type strain PG2. For complementation, *pdhAB* was cloned downstream of a strong *vpma* promoter and upstream of a *lacZ* reporter gene in a newly constructed complementation vector. When transformed with this vector the *pdhB *mutant recovered its normal growth and colony morphology. Interestingly, the *pdhB* mutant also had significantly reduced invasiveness in HeLa cells, as revealed by double immunofluorescence staining. This deficiency was recovered in the complemented strain, which had invasiveness comparable to that of PG2. Taken together, these data indicate that pyruvate dehydrogenase might be an important player in infection with and colonization by *M*. *agalactiae*.

## Introduction

Mycoplasmas are the simplest self-replicating organisms and have evolved from Gram-positive bacteria through reductive evolution [1, 2]. As a result of their limited biosynthetic and metabolic capabilities, mycoplasmas rely on infected host cells for their nutrition and thus lead a parasitic life [3, 4]. As successful pathogens, they have developed mechanisms to invade and survive within host cells. Surface lipoproteins play an important role in the adhesion and invasion of mycoplasmas and also in evading the host immune response because of their variable sequence and expression [5, 6]. Apart from surface lipoproteins, some metabolic enzymes are also known to play a role in host-pathogen interactions [7–10]. Although there have been suggestions by some that metabolic enzymes should not be considered as virulence factors [11], several studies have identified and described them as important pathogenicity determinants in addition to their role in basic metabolism [12, 13]. Genomes of mycoplasmas were among the first to be sequenced, but there is a lack of knowledge about their pathogenicity. This dearth of information is mainly attributed to insufficient availability of genetic tools and also because mycoplasmas are not so amenable to genetic manipulation. Additionally, only a few mycoplasmas have self-replicating plasmids that can be used in genetic studies [14–17]. Nevertheless, efforts have been made to establish genetic tools through the development of *oriC*-based shuttle vectors and studies have also been undertaken to genetically manipulate mycoplasmas using conventional methods, such as transposition and homologous recombination [17].

*M*. *agalactiae* is a pathogen of small ruminants and the main etiological agent of contagious agalactia syndrome, which causes major economic losses and is difficult to eradicate because carriers continue to shed and infect new herds for many years after the initial infection [18, 19]. Although the disease characteristics and pathology are well documented, little is known about the pathogenicity determinants of *M*. *agalactiae* or the mechanisms involved in infection and persistence. As a first step towards understanding the pathogenicity of *M*. *agalactiae*, *ori*C-based shuttle vectors were developed and genetic manipulation was achieved using Tn*4001*mod [20, 21]. To understand the role of Vpmas (Variable surface proteins of *M*. *agalactiae*) in pathogenicity, phase locked mutants were generated through the targeted disruption of the site-specific recombinase gene *xer1* [22] and it was shown that Vpmas and Vpma phase variation play a role in the pathogenesis of *M*. *agalactiae* [23]. Other studies have elaborated on interactions with host cells in vitro [24] and in vivo [25], biofilm formation [26] and host cell adhesion [27]. Except for a handful of such reports, and despite the availability of its genome sequence since 2007 [28], functional analysis of *M*. *agalactiae* genes is largely unaccomplished.

**Fig 1:**
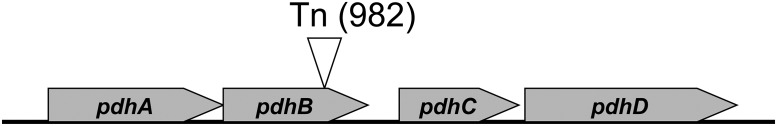
Schematic representation of the pyruvate dehydrogenase (PDH) complex in *M*. *agalactiae* type strain PG2. The nucleotide site for transposon (Tn) insertion in the *pdhB* gene is shown.

Transposon mutagenesis of *M*. *agalactiae* strain PG2 resulted in a clone carrying an insertion in the *pdhB* gene (MAG_0940), which encodes the E1 beta subunit of the pyruvate dehydrogenase (PDH) complex, which converts pyruvate to acetyl-CoA (Fig. 1). The *M*. *agalactiae* PDH complex consists of 4 genes arranged in 2 operons, designated *pdhAB* and *pdhCD*, encoding the E1 and E2 subunits, respectively. The aim of this work was to study the effect of *pdhB* gene disruption on the in vitro growth characteristics of *M*. *agalactiae*. Role of PDHB has previously been reported in the host cell interactions of other mycoplasma species [7, 8]. In this context, we wanted to check the *pdhB* mutant for its ability to invade mammalian cells. Overall, the data indicate that the PDH complex not only plays a conventional role in *M*. *agalactiae’s* energy metabolism but also decreases its invasion capacity, which in turn might have an effect on its pathogenesis.

## Material and Methods

### Bacterial cultures and growth conditions

The pathogenic *M*. *agalactiae* type strain PG2 [28, 29] and the *pdhB* transposon mutant were grown at 37°C in SP4 medium supplemented with penicillin, pyruvate, and phenol red as an indicator, as described before [21]. Gentamicin sulphate (50 μg/ml) was added to both broth and agar plates for the propagation of the *pdhB* transposon mutant. *E*. *coli* DH10B (Invitrogen GmbH, Lofer, Austria) transformants containing the transposon vector pISM2062 or the *M*. *agalactiae oriC* vector pMM21–7 were grown in LB broth (10 g tryptone, 5 g yeast extract, and 5 g of NaCl per litre) supplemented with ampicillin (100 μg/ml), gentamicin sulphate (7 μg/ml) or tetracycline (100 μg/ml). The *M*. *agalactiae pdhB* mutant containing the complementation plasmid pP*lacZ* was grown in SP4 broth containing both tetracycline (2 μg/ml) and gentamicin (50 μg/ml). To monitor *lacZ* expression on the basis of blue-white colonies, *M*. *agalactiae* clones were grown on SP4 agar supplemented with 5-bromo-4-chloro-3-indolyl-β-D-galactopyranoside (X-Gal) at a concentration of 160 μg/ml [30].

### Transposon mutant library

Transposon mutants were generated in *M*. *agalactiae* type strain PG2 using the suicide plasmid pISM2062, containing transposon Tn*4001*mod [31], as described previously [21]. Transposon insertion sites were sequenced using a novel method based on ligation mediated Quantitative Target Display (QTD) PCR [32]. This was further modified and standardized for the *M*. *agalactiae* genome to sequence the Tn mutant library [33].

### DNA manipulations

Preparation of plasmids and genomic DNA and the isolation of DNA fragments from agarose gels were performed using kits from Promega (Wizard SV gel and PCR clean-up system, Promega, Mannheim, Germany) and Qiagen (QIAamp DNA Mini kit; Qiagen GmbH, Hilden, Germany). Restriction endonucleases and modifying enzymes were purchased from Promega and Fermentas Life Science and Antarctic phosphatase was purchased from New England Biolabs (Frankfurt am Main, Germany). Transformation of *E*. *coli* cells was performed by electroporation with a Bio-Rad Gene-Pulser II (Bio-Rad Laboratories GmbH, Vienna, Austria) at settings of 1.25 V, 25 F, and 200 Ω. Transformation of *M*. *agalactiae* cells was also carried out by electroporation as described previously [21]. Oligonucleotide synthesis and sequencing was carried out at Microsynth AG (Balgach, Switzerland) and Agowa Sequencing Service (LGC's AGOWA Genomics, Berlin, Germany), respectively. Standard molecular procedures were performed as described earlier [34].

### *In silico* analysis

Promoters in mycoplasmas are not well defined. Usually promoter regions lie upstream of the transcriptional start site, although the positions of the conventional-10 and -35 elements may vary slightly. In order to find the putative *vpma* promoter region, a 400 bp sequence upstream of *vpmaY* was retrieved from NCBI and analysed using Neural Network Promoter Prediction (NNPP) (http://www.fruitfly.org/seq_tools/promoter.html) and BPRO (http://linux1.softberry.com/berry.phtml?topic=bprom&group=programs&subgroup=gfindb). The highest score fragments predicted by both these programs were selected for cloning.

### PCR amplification

PCR amplification of *pdhAB* and the *vpma* promoter region was carried out in a total volume of 50 μl using 200 ng of PG2 genomic DNA as template. The reaction mixture contained 1 μM of the respective primers (Table 1), 25 mM MgCl_2_, 0.2 μM of each deoxynucleoside triphosphate (dNTP), and 5 U of *Taq* DNA polymerase (Promega) in 1× PCR buffer supplied by the manufacturer. Cycling parameters consisted of initial denaturation at 3 min at 94°C, followed by 30 cycles of 1 min of denaturation at 94°C, 1 min of annealing at 54°C and either 1 min (*vpma* promoter) or 2 min (*pdhAB*) at 72°C for extension, with a final extension step of 5 min at 72°C. The PCR products were either gel extracted or purified with the appropriate kit before proceeding to restriction endonuclease digestion.

**Table 1:** List of oligonucleotides used in this study.

Name	Sequence
PromfpB	AGT CAA GGA TCC TCT TCA CGA TTC TGT T
PromrpN	CTC TAT CCA TGG CGC TAC AAT CTT CTA CT
pdhABfpN	ACT TAT CCA TGG CAG CTA GTC CGC TTA
pdhABrfN	GTA CTT CCA TGG CTC CGT ACG GTT ATA

Underlined sequences represent incorporated restriction sites.

### Plasmid construction

#### Construction of p*lacZ* and pP*lacZ*

The plasmid p*lacZ* was generated by cloning the *lacZ* reporter gene between the *Bam*HI and *Sma*I sites in the pMM21–7 shuttle vector containing *M*. *agalactiae oriC* [20]. The pIL plasmid [30] containing *lacZ* was digested with *Bam*HI and *Sma*I and ligated into the vector pMM21–7, which had been digested with the same enzymes. The resulting p*lacZ* construct was used to transform *M*. *agalactiae* PG2 to check *lacZ* expression in absence of a promoter and found that there were no blue colonies expressing *lacZ*. The p*lacZ* plasmid was further developed into a complementation vector by cloning a strong *M*. *agalactiae* promoter in it. The putative *vpma* promoter region was amplified from *M*. *agalactiae* strain PG2 using the primers PromfpB and PromrpN (Table 1), which contained *Bam*HI and *Nco*I sites, respectively and this PCR fragment was introduced into corresponding sites in p*lacZ*. The resulting 12.6 kb plasmid was designated pP*lacZ* and introduced into *M*. *agalactiae* PG2 to check for *lacZ* expression in presence of the cloned putative promoter.

#### Construction of pP*pdhABlacZ*

The complementation vector pP*lacZ* containing the *vpma* promoter was used to clone *pdhB* gene. However, our repeated attempts to clone the *pdhB* gene alone in pP*lacZ* were unsuccessful. Hence, it was decided to clone *pdhAB*, as *pdhA* and *pdhB* are found in tandem in the same operon. The 2.5 kb region corresponding to *pdhAB* was amplified with primers pdhABfpN and pdhABrfN, which contained *Nco*I cleavage sites (see Table 1) and the PCR fragment was ligated into pP*lacZ* after digestion with *Nco*I to generate pP*pdhABlacZ*. The resulting construct was analyzed to determine the orientation of the *pdhAB* gene, and this was also verified by sequencing before it was used to transform the *pdhB* mutant. *M*. *agalactiae* clones containing this plasmid were further examined to confirm the presence of *pdhAB* via Southern blot analysis using a 2.5 kb DIG-labeled *pdhAB* PCR fragment as a probe (Roche) as described previously [20].

### Growth curve analysis

Growth of the *pdhB* mutant in SP4 medium was compared with the wild type strain PG2 at 37°C. Both the cultures were grown for 48 h with appropriate antibiotic in SP4 medium and growth was initiated by subculturing 1:100 dilutions of the cultures in SP4 medium in duplicate. Starting concentrations of all the cultures were determined by plating suitable dilutions on SP4 agar plates. At 6, 12, 24 and 48 h post inoculation, small aliquots of each culture were diluted in SP4 medium and plated on SP4 agar plates. After 5–6 days of incubation at 37°C, mycoplasma colonies were counted under an inverted microscope to determine concentrations at each time point. The growth curves were subsequently determined a second time together with the complemented *pdhB* mutant. The latter growth curves were performed three times in duplicate.

### *M*. *agalactiae* cell invasion

Invasion into HeLa-229 (ATCC CCL-2.1) cells was assessed by infecting them for 24 h with wild type PG2, mutant (Δ*pdhB*) and complemented (Δ*pdhB*:: pP*pdhABlacZ*) strains as described previously [25] using double immunofluorescence (DIF). Invasion was quantified by randomly selecting and analyzing about hundred HeLa cells in random microscopic fields. The results were expressed as the average number of invading mycoplasmas per 100 HeLa cells. The experiments were performed in duplicate for at least four different times.

### Statistical analysis

The results of growth curves performed at three different times in duplicate were expressed as mean ± standard deviation (SD) of *n* independent values. The invasion experiments were performed at least four times in duplicate. The significance of differences between the means of the experiments was calculated by Student’s *t* test using GraphPad Prism 5 (Graphpad Software Inc, CA, USA). Differences with *P<* 0.05 were considered significant.

## Results

### *pdhB* transposon mutant exhibits slow growth during logarithmic phase and altered colony size and morphology

**Fig 2:**
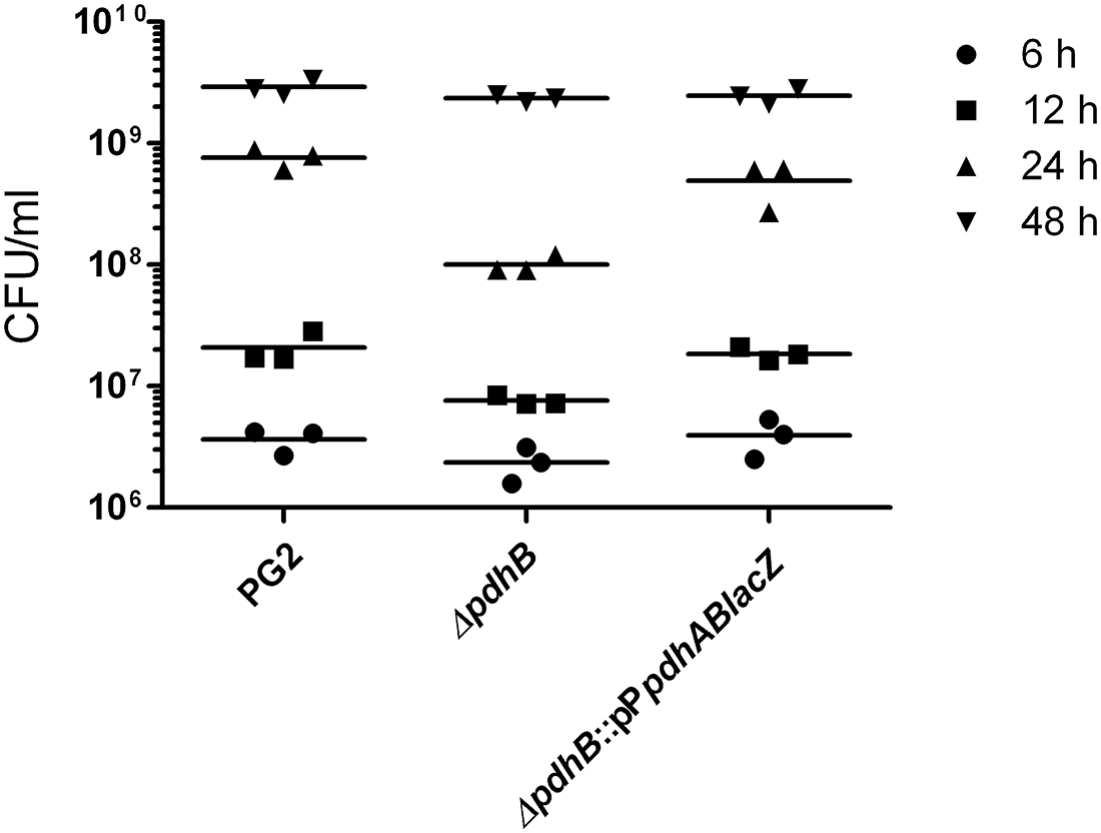
*pdhB* affects *M*. *agalactiae* growth *in vitro*. Dot plot representation of growth curve analysis of wild type PG2, Δ*pdhB* mutant and complemented strain Δ*pdhB*:: pP*pdhABlacZ* at 6 (circles), 12 (squares), 24 (triangles) and 48 (inverted triangles) hours in SP4 medium. Each point represents a single experiment done in duplicates.

Transposon mutants of *M*. *agalactiae* were checked for *in vitro* growth defects, and the results demonstrated slower growth of the *pdhB* mutant compared to the wild type strain PG2. This mutant carried a transposon insertion at the nucleotide position 982 of the *pdhB* gene (MAG_0940), which was confirmed by sequencing. As illustrated in Fig. 1, *pdhB* gene in *M*. *agalactiae* type strain PG2 is found in tandem with *pdhA* on the *pdhAB* operon that encodes the E1 subunit of pyruvate dehydrogenase (PDH) complex. A significantly slower growth rate (P<0.05) was observed for the *pdhB* mutant during the log phase (Fig. 2). A growth difference of nearly two-fold was observed at 6 h between the mutant and wild type strains, which further increased to a four-fold difference at 12 h (Fig. 2). At 24 h the growth difference was nearly 8 fold (Fig. 2), indicating the inability of the mutant to efficiently replicate during the prolific logarithmic growth phase. However, the mutant was able to recover its growth to that of wild type strain during the stationary phase at 48 h (Fig. 2).

**Fig 3:**
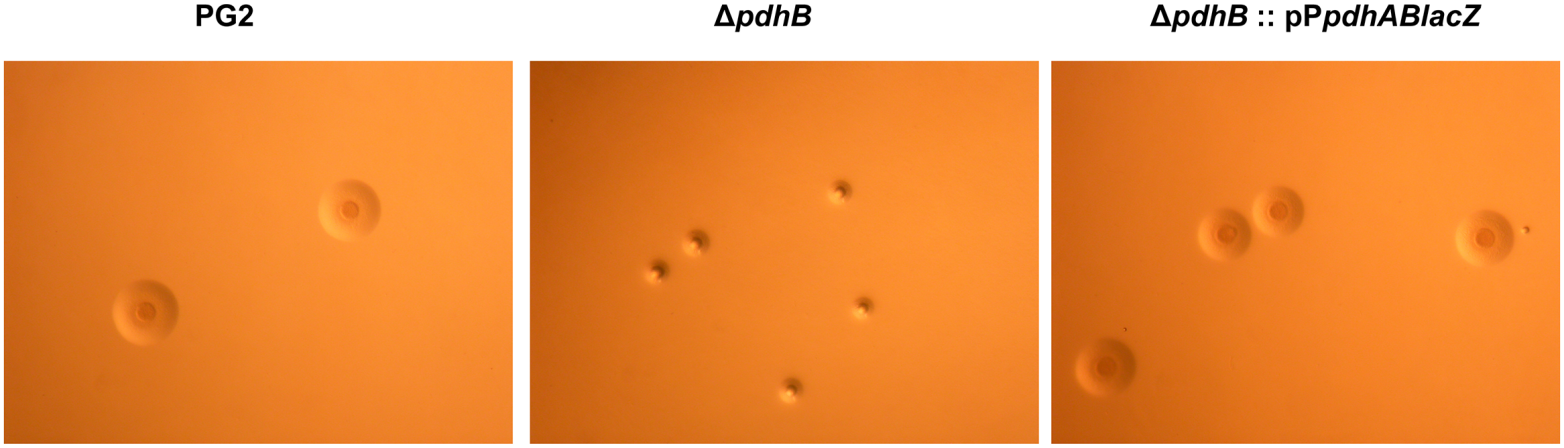
Effect of *pdhB* disruption on colony morphology of *M*. *agalactiae*. Representative colonies of wild type PG2, mutant (Δ*pdhB*) and complemented (Δ*pdhB*:: pP*pdhABlacZ*) strain after 5 days of parallel growth on SP4 agar at 37°C. All three micrographs were made using Stereo microscope BMS 74955 at the same time using the same settings and magnification (x3).

The transposon insertion in the *pdhB* gene also led to a smaller colony size compared to the wild type counterpart, even when plated on rich SP4 agar medium (Fig. 3). In addition, the *pdhB* mutant colonies did not have the “fried-egg” morphology, with most colonies showing just the dark dense central “down growth” button without the peripheral surface growth when observed under an inverted microscope [35] (Fig. 3).

### *pdhB* mutant is deficient in HeLa cell invasion

**Fig 4:**
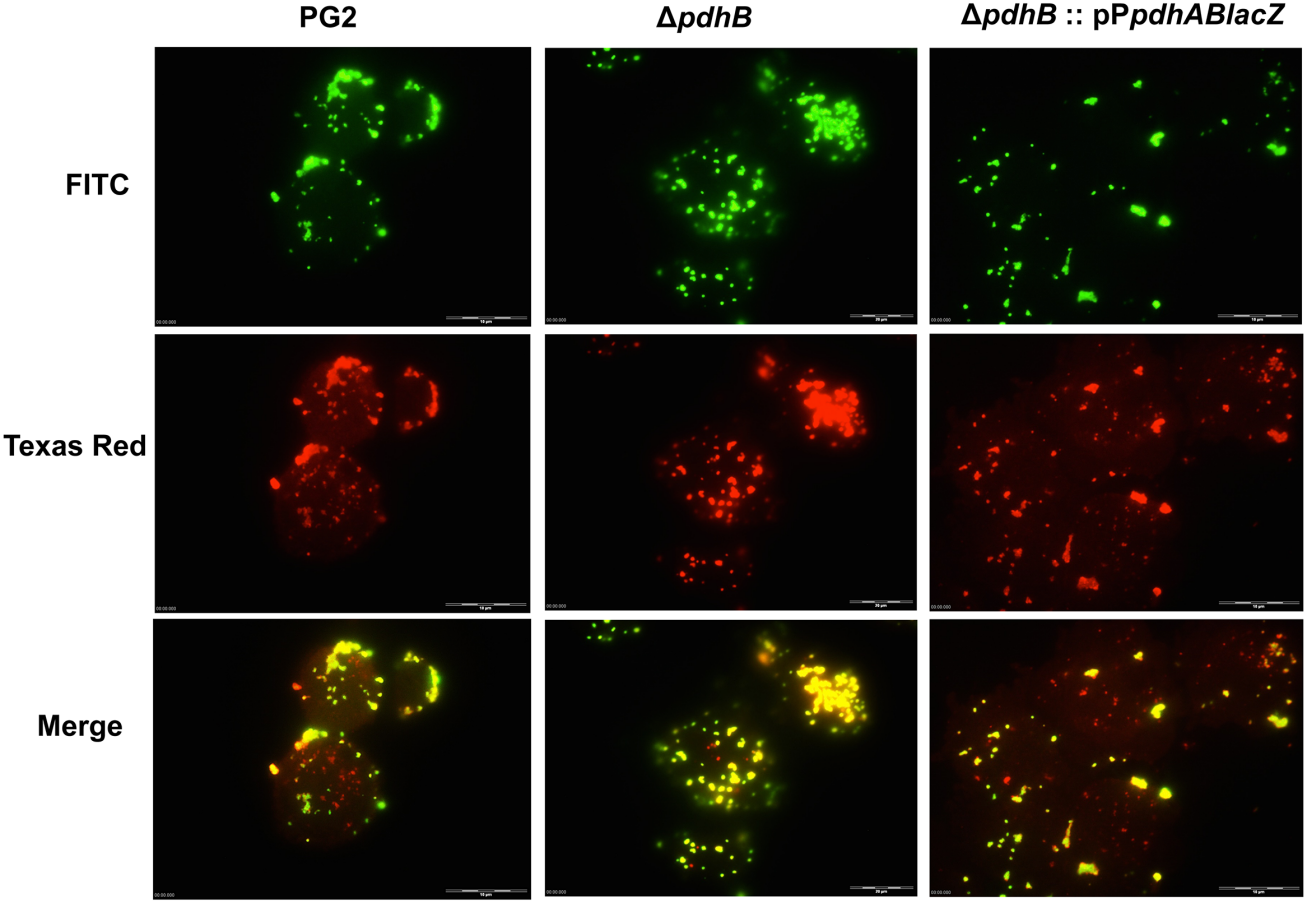
Micrographs depicting the interaction of *M. agalactiae* with HeLa-229 cells. Double immunofluorescence staining showing the invasion of the wild type PG2 strain, the *pdhB* mutant (Δ*pdhB*) and the complemented strain (Δ*pdhB*:: pP*pdhABlacZ)* of the *pdhB* mutant into HeLa cells. The three panels of each strain correspond to the same area of the infected monolayer. FITC fluorescence showing green colored extracellular mycoplasmas and Texas Red fluorescence showing extracellular and intracellular mycoplasmas. Merged images indicating the localization of extracellular (yellow) and intracellular (red) mycoplasmas. Bars, 10 μm.

**Fig 5:**
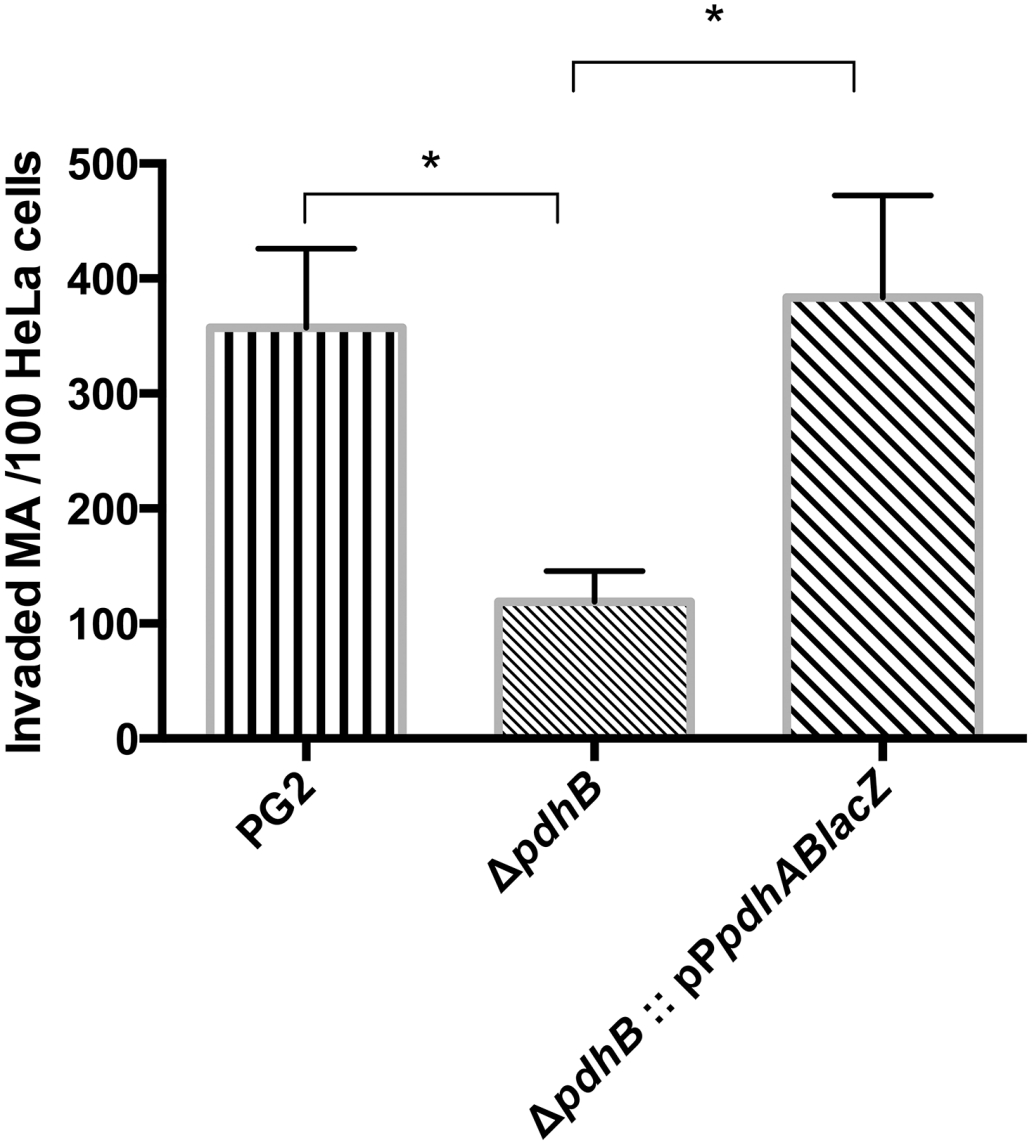
Effect of *pdhB* mutation on HeLa cell invasion analyzed via the double immunofluorence assay. Cellular invasion was evaluated using the wild type *M*. *agalacatie* PG2 strain, the *pdhB* mutant (Δ*pdhB*) and the complemented strain (Δ*pdhB*:: pP*pdhABlacZ*) by counting the internalized mycoplasmas using an Olympus AX-70 epifluorescence microscope. The average number of internalized *M*. *agalactiae* (MA) per 100 HeLa cells was determined using 100 randomly selected microscopic fields. The bars and error bars indicate the means and standard deviations of triplicate experiments (* P<0.05).

A double immunofluorescence assay was performed to evaluate the invasiveness of the *pdhB* mutant in comparison with the wild type PG2 strain. Extracellular mycoplasmas were stained with FITC conjugated antibody, while Texas red was used to stain both intracellular and extracellular bacteria. After merging the two images the intracellular mycoplasmas appeared red in color, while the extracellular organisms were yellow (Fig. 4). Observation of HeLa cells infected with *pdhB* mutant revealed few or no intracellular mycoplasmas in most microscopic fields (Fig. 4) whereas the HeLa cells infected with the wild type PG2 contained large numbers of intracellular bacteria (Fig. 4). There were significantly lower (P<0.05) numbers of invading mutant mycoplasmas compared to the wild type PG2 (Figs. 4 and 5). On average, only 120 intracellular mutant bacteria were observed per hundred HeLa cells, whereas the number of intracellular wild type PG2 cells was nearly three-fold higher (357 mycoplasma cells/100 HeLa cells) (Fig. 5). Hence, the *pdhB* mutation led to a significant reduction in the ability of *M*. *agalactiae* to invade non-phagocytic eukaryotic cells.

The inefficient invasion of the *pdhB* mutant cannot be attributed to its slower growth phenotype because it showed slower growth only in the early logarithmic phase and reached wild type levels during the stationary phase. Both the mutant and the wild type strains were cultured for 48 hours and had reached stationary phase when used to infect HeLa cells. This was also evident from the calculated multiplicity of infection (MOI), which was found to be comparable for both the *pdhB* mutant and wild type PG2. As controls, uninfected eukaryotic monolayers were also stained with FITC and Texas Red to rule out the possibility of any previous contamination with mycoplasmas and/or any cross-reactivity of antibodies (data not shown).

### Complementation studies

#### Generation of reporter based complementation vector

To confirm the role of *pdhB* in the in vitro growth of *M*. *agalactiae*, the altered colony morphology and in invasion of eukaryotic cells, the wild type *pdhAB* genes were cloned into a complementation vector pP*lacZ* that was constructed as described above. PG2 genome contains a single copy of *pdhB* gene in tandem with *pdhA*, and both genes are likely to be expressed together as a strong promoter like element is found upstream of *pdhA* gene. Since our efforts to clone the *pdhB* gene were unsuccessful, *pdhAB* genes were cloned in pP*lacZ* for complementation. To ensure expression of the cloned genes a strong promoter was required. It was decided to clone the putative *vpma* promoter as Vpmas are one of the most abundantly expressed proteins of *M*. *agalactiae* [36]. The vector was generated by cloning a strong putative promoter upstream of the *lacZ* gene. The putative promoter region upstream of the *vpmaY* gene was analyzed to predict the promoter using BRPOM and NNPP. NNPP identified ten fragments in the 400 bp region upstream of *vpmaY* at a cut-off score of 0.8. A sequence 273–300 bp upstream of *vpmaY* had the highest possible score of 1 (Table 2). BPROM also predicted possible-10 and -35 elements in the same region (Table 2). This 400 bp region upstream of *vpmaY* was PCR amplified and cloned as a putative strong promoter in front of the *lacZ* gene in the autonomously replicating vector p*lacZ* as described above. The cloned *vpma* promoter showed strong activity, as indicated by blue colored colonies when pP*lacZ* was introduced into *M*. *agalactiae* PG2 (S1 Fig.). In the absence of this promoter, the *lacZ* gene alone in p*lacZ* failed to induce formation of blue colonies (S1 Fig.). Hence, *pdhAB* was cloned in the complementation vector pP*lacZ* to generate pP*pdhABlacZ*. Interestingly, pP*pdhABlacZ* also induced formation of faint blue-colored colonies when introduced into the *pdhB* mutant (S1 Fig.), indicating the strong functional activity of the cloned *vpma* promoter. The pP*pdhABlacZ* construct was sequenced to confirm the orientation and correct sequence of *pdhAB*. The plasmid was then introduced into the *pdhB* mutant to generate the complemented strain Δ*pdhB*:: pP*pdhABlacZ*, in which the presence of intact *pdhAB* gene was verified by Southern blot analysis depicting autonomous replication, as well as chromosomal integration of the complementation plasmid at the mutated *pdhAB* locus (S2 Fig.).

**Table 2:** Promoter regions upstream of the *vpmaY* gene predicted by two different promoter prediction programs.

Input sequence	Start	End	Score	Promoter sequence	Software
-400 bp of vpmaY	273	318	1.00	acaaaaaatttttgctattttttttttttttatataatattgtcatctat	NNPP
	269	318	1.00	caaaaaatttttgctattttttttttttttatataatattgtcatctatt -35 -10	BPROM

#### Complementation of *pdhB* mutant phenotypes

Growth curve analysis clearly demonstrated the rescue of normal growth in the complemented strain Δ*pdhB*:: pP*pdhABlac*Z, which had a log phase growth comparable to that of the wild type PG2 strain (Fig. 2). In addition *pdhAB* complementation of the mutant restored its colony size and its morphology to the normal “fried-egg” phenotype (Fig. 3).

Similarly, significantly higher numbers of the complemented Δ*pdhB*:: pP*pdhABlac*Z strain (P<0.05) could invade HeLa cells compared to the *pdhB* mutant (Fig. 5), with 387 intracellular mycoplasmas seen per 100 HeLa cells infected with the complemented strain (Fig. 5). Although the counts were slightly higher than for the wild type PG2 strain (357 mycoplasmas/100 HeLa cells), they were not significantly different (P>0.05).

Altogether, these data clearly demonstrate that the growth and invasion deficits, as well as the altered colony morphology of the *pdhB* mutant were due to the absence of the E1 beta subunit of the pyruvate dehydrogenase complex and that these phenotypic changes were reversed by the introduction of intact *pdhAB* via complementation plasmid pP*pdhABlacZ*.

## Discussion

Transposon mutagenesis is a powerful tool to identify genetic factors involved in the virulence of pathogenic bacteria. It has also been widely used in mycoplasmas to gain an understanding of the role of different genes involved in motility, cytadherence and restriction modification systems [37–39]. After successful manipulation of the *M*. *agalactiae* genome using Tn*4001*mod, a library of mutants was created and all the disrupted genes were sequenced [21, 33]. Several important loci belonging to different functional categories such as lipoproteins, transporters and metabolic enzymes, were found to have been disrupted. Among the several loci belonging to the latter category was the *pdhB* gene, encoding the E1β subunit of the PDH complex.

**Fig 6:**
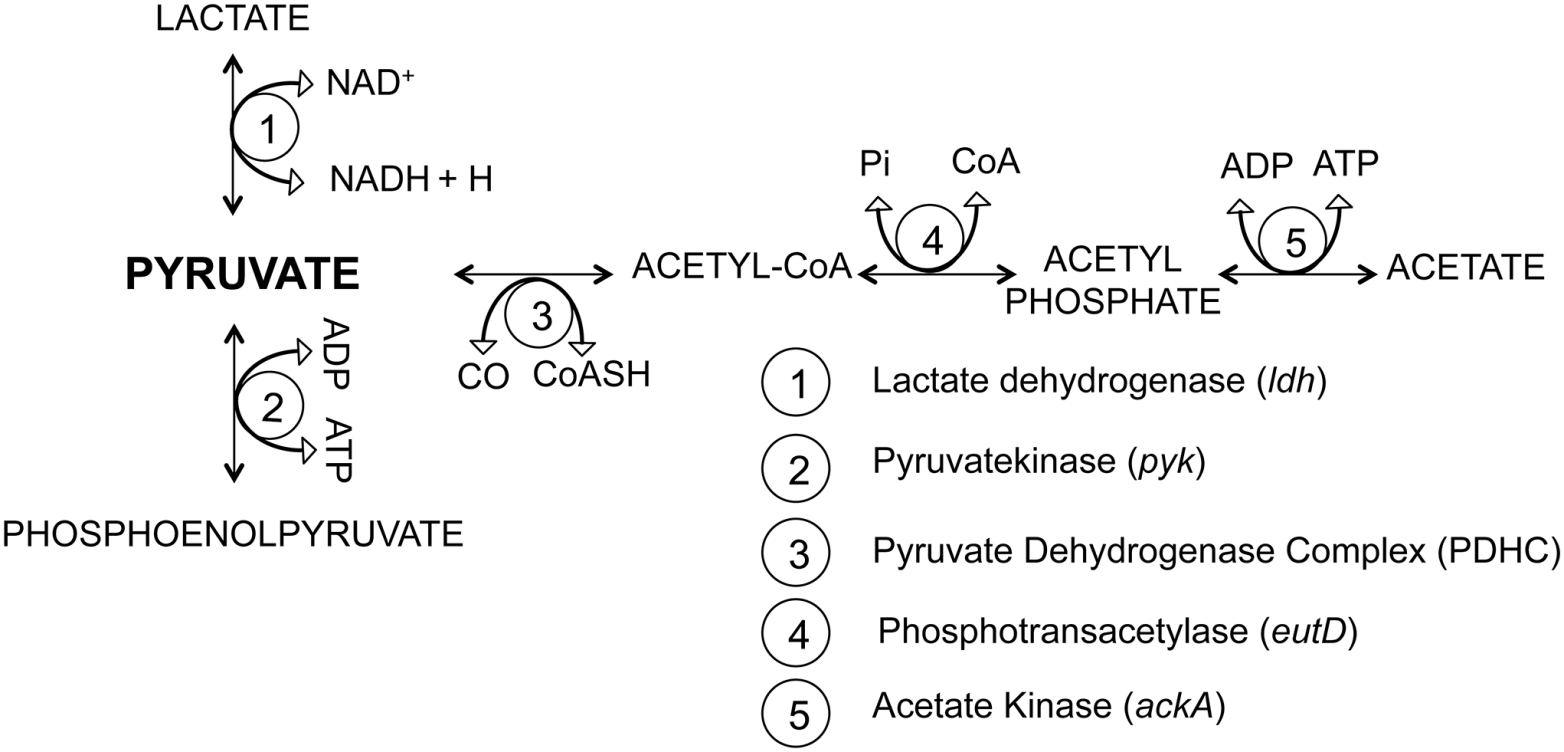
Schematic representation of alternative pathways for pyruvate metabolism in *M*. *agalactiae*.

Here, we have studied the effect of *pdhB* disruption on *M*. *agalactiae’s* growth and invasion of host cells. This gene is part of the PDH multi-enzyme complex involved in the conversion of pyruvate to acetyl-CoA. It has been demonstrated previously that growth of *M*. *agalactiae* is markedly increased in the presence of pyruvate [40], suggesting that degradation of pyruvate is a major energy yielding pathway. Pyruvate is known to be metabolized to acetate in a series of reactions involving the PDH complex, phosphotransacetylase (EutD) and acetate kinase (AckA), with the generation of ATP. Although any defect in this pathway is likely to affect growth, it is interesting to note that unlike the *pdhB* mutant, transposon insertion in the *eutD* gene did not cause any growth retardation or altered colony morphology (data not shown). Our results with the *pdhB* mutant suggest that conversion of pyruvate to acetyl-CoA is critical and its absence caused the slow log phase growth and altered colony morphology in the *M*. *agalactiae pdhB* mutant. The growth of the *pdhB* mutant recovered during the stationary phase, indicating the involvement of alternative pyruvate breakdown pathways operational at this stage (Fig. 6). It could be hypothesized that during the prolific log phase growth, the *pdhB* mutant is unable to metabolize pyruvate to meet its energy needs and as a result pyruvate accumulates, which is then likely to slow down the other pyruvate metabolizing pathways, if operational, due to reverse kinetics. During the stationary phase, perhaps an alternative pathway becomes predominant (Fig. 6), namely conversion of pyruvate to lactate via lactate dehydrogenase, and hence the recovery of growth by the *pdhB* mutant. This study highlights the fact that, despite the many alternative pathways for (pyruvate) metabolism in *Mollicutes*, and despite the very rich growth medium, the presence or absence of specific gene products can significantly influence the growth rate of these pathogens. Such variation in metabolic activity may have consequences for pathogenesis, and the survival and propagation of these pathogens.

It is also interesting to note that the *pdhB* mutation also has an adverse effect on *M*. *agalactiae’s* ability to invade HeLa cells. Involvement of mycoplasma glycolytic enzymes in host cell interactions has been reported previously [7, 10, 41]. Among them are α-enolase, GAPDH and PDHB, which were shown to be involved in interactions with host extracellular matrix proteins [7, 8, 10, 41–43]. Since adhesion is a prerequisite for invasion into host cells, it is likely that these proteins also play a role in internalization of bacteria. Although our results revealed a reduced capacity of the *pdhB* mutant to invade HeLa cells, we did not find any adverse effect of the *pdhB* mutation on the cell adhesion phenotype of the mutant compared to the wild type strain PG2 as both exhibited comparable adhesion capacities. This indicates that pyruvate dehydrogenase likely acts at a stage secondary to initial contact for internalization into eukaryotic cells. Confirmation of the role of pyruvate dehydrogenase in cell invasion was substantiated by complementation studies. To our knowledge this is the first report describing the role of pyruvate dehydrogenase in host cell invasion and further studies might help elaborate the exact details of this process.

Even though PDH mutants have slower *in vitro* growth phenotypes, they have often been detected in STM screens, and PDH has been suggested to play important roles in the pathogenicity of other bacterial pathogens [44, 45]. Recently, PDHB of *M*. *bovis*, which has 98% sequence identity with its *M*. *agalactiae* counterpart, was identified as a novel immunogenic target for a serodiagnostic ELISA [46]. As intracellular invasion by pathogens is believed to play a significant role in their systemic spread and pathogenicity [25, 47–49], it is anticipated that pyruvate dehydrogenase might play a role in *M*. *agalactiae* infections.

## Supporting Information

S1 Fig Schematic representation of plasmids and *M*. *agalactiae* transformant colonies. (A) pP*lacZ* with the *lacZ* gene alone failed to show expression of LacZ (white colonies), (B) pP*lacZ* carrying the *vpma* promoter in front of the *lacZ* gene led to intense blue colored colonies, (C) pP*pdhABlacZ* carrying the *pdhAB* genes cloned between the *vpma* promoter and the *lacZ* gene showed intermediate blue colored colonies, thereby indicating strong promoter activity.
(TIF)

S2 Fig Southern blot analysis. Southern blot hybridization of *pdhAB*-complemented Δ*pdhB* showing the free and integrated pP*pdhABlacZ* at the chromosomal *pdhAB* locus. *Eco*RI digested DNA of the complementation clone Δ*pdhB*:: pP*pdhABlacZ* (lane 5) is shown in comparison with wt strain PG2 (lane 2), Δ*oppC* (lane 3), Δ*pdhB* (lane 4) and complementation plasmid pP*pdhABlacZ* (lane 6) after hybridization with *pdhAB*-specific DIG-labelled probe. C corresponds to the 2.1 kb chromosomal fragment seen in PG2 and Δ*oppC* (transposon mutant) control whereas M corresponds to the 6.6 kb mutated *pdhAB* fragment seen in Δ*pdhB* and complemented strain. A single putative homologous recombination event between the *pdhAB* genes carried by the complementation plasmid pP*pdhABlacZ* and the mutated chromosomal *pdhAB* region leads to the integration of *pPpdhABlacZ* into the chromosome and specifically identifies a 9.5 kb chromosomal integration fragment CI in the complementation clone. P represents the 10.6 kb fragment corresponding to the free-replicating complementation plasmid as seen in lanes for the complementation clone and pP*pdhABlacZ* plasmid. λ-*Hind*III DNA size marker (lane1).
(TIF)
