# Correction: Disruption of the *pdhB* Pyruvate Dehydrogenase Gene Affects Colony Morphology, *In Vitro* Growth and Cell Invasiveness of *Mycoplasma agalactiae*

**DOI:** 10.1371/journal.pone.0131134

**Published:** 2015-06-19

**Authors:** The PLOS ONE Staff

## Notice of Republication

This article was republished on May 26, 2015, to correct an error in the title: “Dehydrogenase” was misspelled “Dehyrogenase.” Please download this article again to view the correct version. The originally published, uncorrected article and the republished, corrected article are provided here for reference.

## Supporting Information

S1 File Originally published, uncorrected article. (PDF)

S2 File Republished, corrected article. (PDF)
