# Supplementary figures and images for: Disruption of the pdhB Pyruvate Dehydrogenase Gene Affects Colony Morphology, In Vitro Growth and Cell Invasiveness of Mycoplasma agalactiae
Source: PLoS One. 2015 Mar 23;10(3):e0119706. doi: 10.1371/journal.pone.0119706 (PMC4370745; doi:10.1371/journal.pone.0119706)

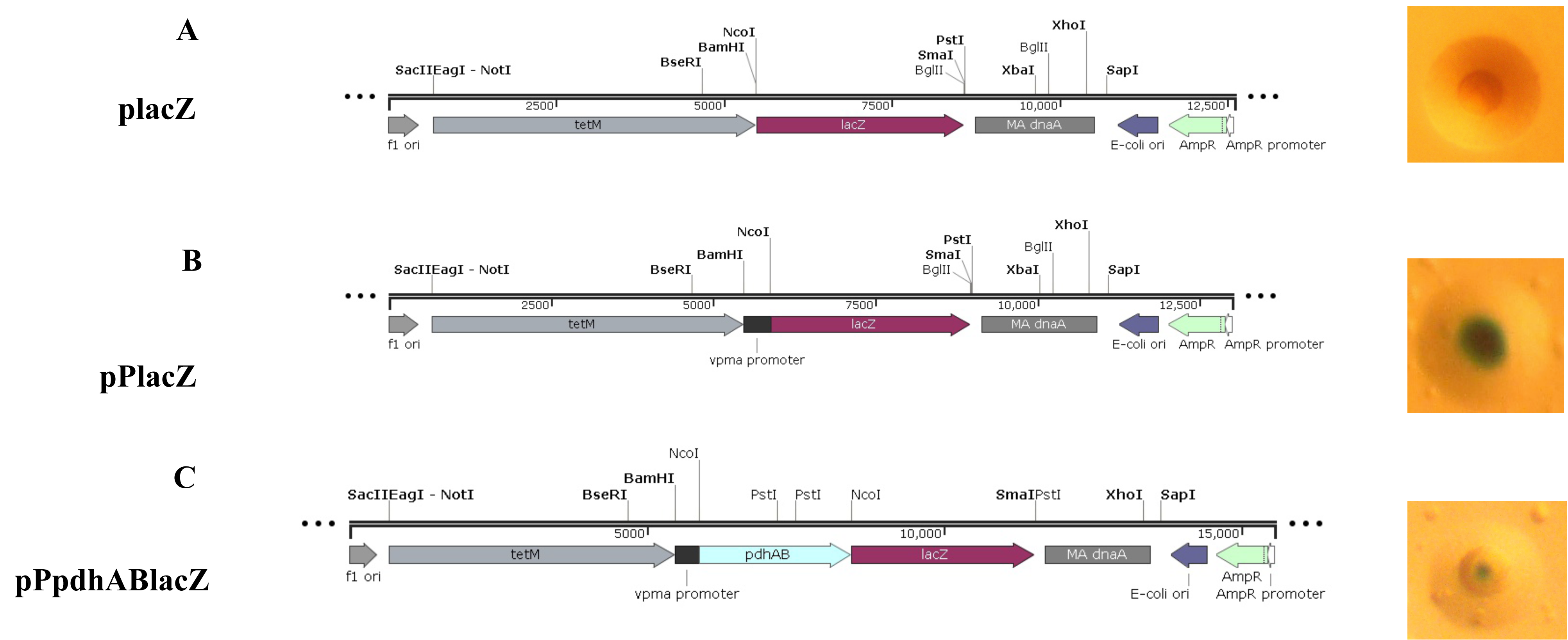

Supplement: S1 Fig — (A) pPlacZ with the lacZ gene alone failed to show expression of LacZ (white colonies), (B) pPlacZ carrying the vpma promoter in front of the lacZ gene led to intense blue colored colonies, (C) pPpdhABlacZ carrying the pdhAB genes cloned between the vpma promoter and the lacZ gene showed intermediate blue colored colonies, thereby indicating strong promoter activity. (TIF) [file pone.0119706.s001.tif]

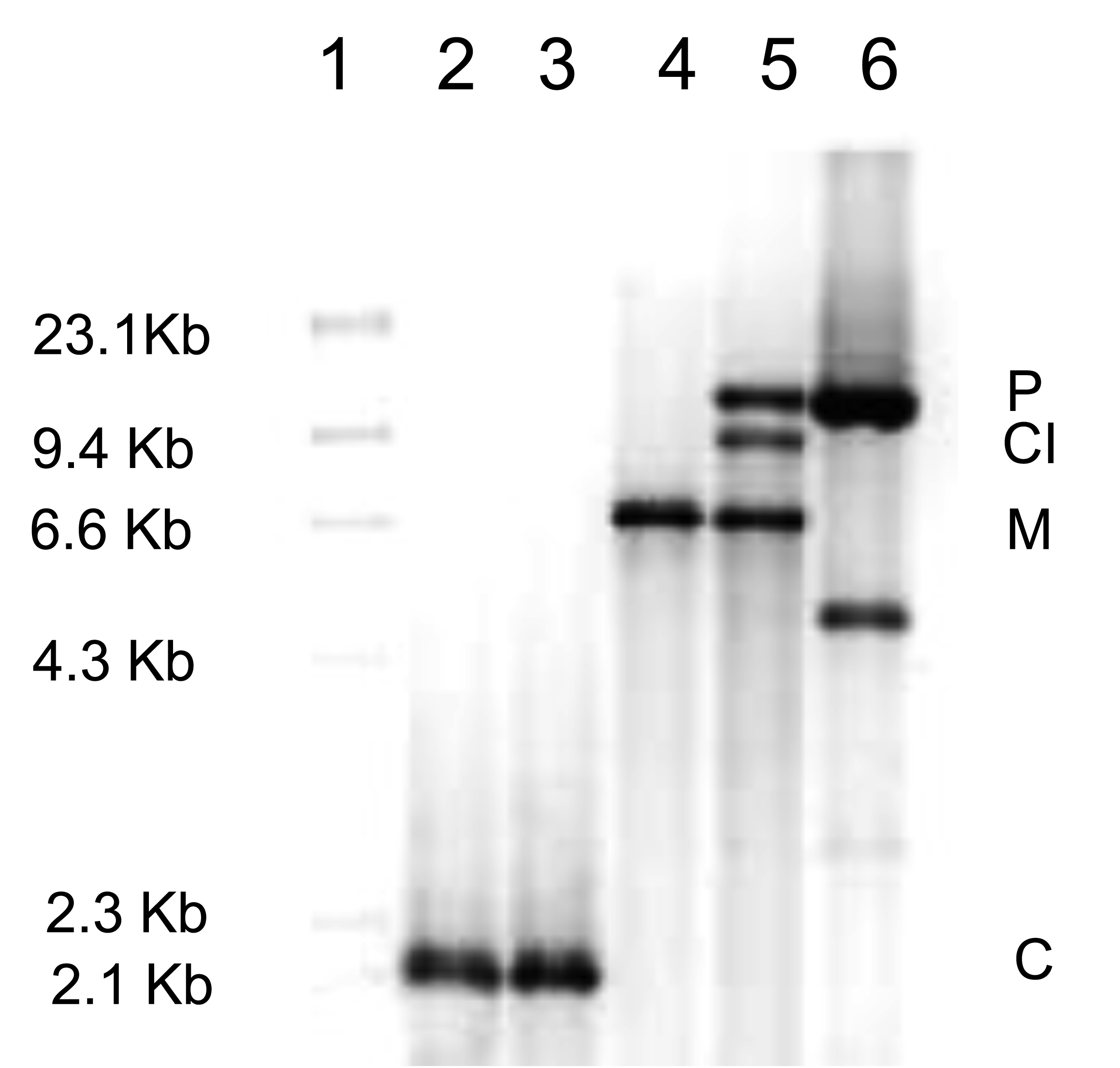

Supplement: S2 Fig — Southern blot hybridization of pdhAB-complemented ΔpdhB showing the free and integrated pPpdhABlacZ at the chromosomal pdhAB locus. EcoRI digested DNA of the complementation clone ΔpdhB:: pPpdhABlacZ (lane 5) is shown in comparison with wt strain PG2 (lane 2), ΔoppC (lane 3), ΔpdhB (lane 4) and complementation plasmid pPpdhABlacZ (lane 6) after hybridization with pdhAB-specific DIG-labelled probe. C corresponds to the 2.1 kb chromosomal fragment seen in PG2 and ΔoppC (transposon mutant) control whereas M corresponds to the 6.6 kb mutated pdhAB fragment seen in ΔpdhB and complemented strain. A single putative homologous recombination event between the pdhAB genes carried by the complementation plasmid pPpdhABlacZ and the mutated chromosomal pdhAB region leads to the integration of pPpdhABlacZ into the chromosome and specifically identifies a 9.5 kb chromosomal integration fragment CI in the complementation clone. P represents the 10.6 kb fragment corresponding to the free-replicating complementation plasmid as seen in lanes for the complementation clone and pPpdhABlacZ plasmid. λ-HindIII DNA size marker (lane1). (TIF) [file pone.0119706.s002.tif]
